# Effects of Remimazolam vs. Sevoflurane Anesthesia on Intraoperative Hemodynamics in Patients with Gastric Cancer Undergoing Robotic Gastrectomy: A Propensity Score-Matched Analysis

**DOI:** 10.3390/jcm11092643

**Published:** 2022-05-08

**Authors:** Bahn Lee, Myoung Hwa Kim, Hee Jung Kong, Hye Jung Shin, Sunmo Yang, Na Young Kim, Dongwoo Chae

**Affiliations:** 1 Department of Anaesthesiology and Pain Medicine, Anaesthesia and Pain Research Institute, Yonsei University College of Medicine, 50-1 Yonsei-ro, Seodaemun-gu, Seoul 03722, Korea; bahnlee@yuhs.ac (B.L.); sarajo8768@yuhs.ac (H.J.K.); sunmong@yuhs.ac (S.Y.); 2 Department of Anaesthesiology and Pain Medicine, Anaesthesia and Pain Research Institute, Yonsei University College of Medicine, Gangnam Severance Hospital, 211 Eonju-ro, Gangnam-gu, Seoul 06273, Korea; kmh2050@yuhs.ac; 3 Biostatistics Collaboration Unit, Yonsei University College of Medicine, 50-1 Yonsei-ro, Seodaemun-gu, Seoul 03722, Korea; hjshin105@yuhs.ac; 4 Department of Pharmacology, Yonsei University College of Medicine, 50-1 Yonsei-ro, Seodaemun-gu, Seoul 03722, Korea

**Keywords:** hemodynamic stability, remimazolam anesthesia, robotic gastrectomy, sevoflurane anesthesia

## Abstract

Remimazolam has been suggested to improve the maintenance of hemodynamic stability when compared with other agents used for general anesthesia. This study aimed to compare the effects of remimazolam and sevoflurane anesthesia on hemodynamic stability in patients undergoing robotic gastrectomy. We retrospectively reviewed the electronic medical records of 199 patients who underwent robotic gastrectomy with sevoflurane (*n* = 135) or remimazolam (*n* = 64) anesthesia from January to November 2021. Propensity scores were used for 1:1 matching between the groups. The primary outcome was the difference in use of intraoperative vasopressors between groups. Secondary outcomes included differences in incidence and dose of vasopressors, as well as intraoperative hemodynamic variables, between groups. Remimazolam anesthesia was associated with a significantly less frequent use of ephedrine (odds ratio (OR): 0.13; 95% confidence interval (CI): 0.05–0.38, *p* < 0.001), phenylephrine (OR: 0.12; 95% CI: 0.04–0.40, *p* < 0.001), and any vasopressor (OR: 0.06; 95% CI: 0.02–0.25, *p* < 0.001) compared with sevoflurane anesthesia. Remimazolam anesthesia enables better maintenance of hemodynamic stability than sevoflurane anesthesia. Thus, remimazolam anesthesia may be beneficial for patients who are expected to experience hypotension due to the combined effects of CO_2_ pneumoperitoneum and the head-up position utilized during robotic gastrectomy.

## 1. Introduction

Robotic gastrectomy has recently attracted attention for its potential to decrease the postoperative complication rates, shorten the duration of postoperative hospitalization, and enhance recovery when compared with conventional open gastrectomy. Several studies have also demonstrated that robotic gastrectomy exhibits a morbidity and mortality profile comparable to that of laparoscopic gastrectomy for patients with gastric cancer undergoing lymph node dissection [1,2,3]. However, some studies have suggested that the procedure is associated with significant hemodynamic instability, mainly due to the combined effects of abdominal insufflation with carbon dioxide (CO_2_) and the need for a prolonged, steep, upward positioning of the head. These essential components of robotic gastrectomy can lead to a decrease in the preload, increase in the systemic vascular resistance, and decrease in the cardiac output [4,5].

Remimazolam, an ultrashort-acting benzodiazepine analogue with a favorable pharmacokinetic profile, has recently been approved for the induction and maintenance of general anesthesia [6,7,8,9]. Remimazolam is unique in that it is rapidly metabolized by liver-bound carboxylesterase and has a context-sensitive half-time of 7.5 min, making it feasible for use in the maintenance of general anesthesia [10]. Among its many advantages, hemodynamic stability has been proposed as the main benefit [6,11].

Although previous studies have reported greater hemodynamic stability in patients receiving remimazolam anesthesia than in those receiving propofol anesthesia [6,11], none have provided evidence regarding remimazolam effects in relation to sevoflurane anesthesia in the context of robotic gastrectomy. In this study, we aimed to compare the effects of remimazolam and sevoflurane anesthesia on intraoperative hemodynamics in patients with gastric cancer undergoing robotic gastrectomy.

## 2. Materials and Methods

### 2.1. Patients

This single-institution retrospective study was approved by the Institutional Review Board and Hospital Research Ethics Committee of Severance Hospital (Yonsei University Health System, Seoul, Korea [protocol number: 4-2021-1611]). The requirement for informed consent was waived given the retrospective study design.

**Figure 1:**
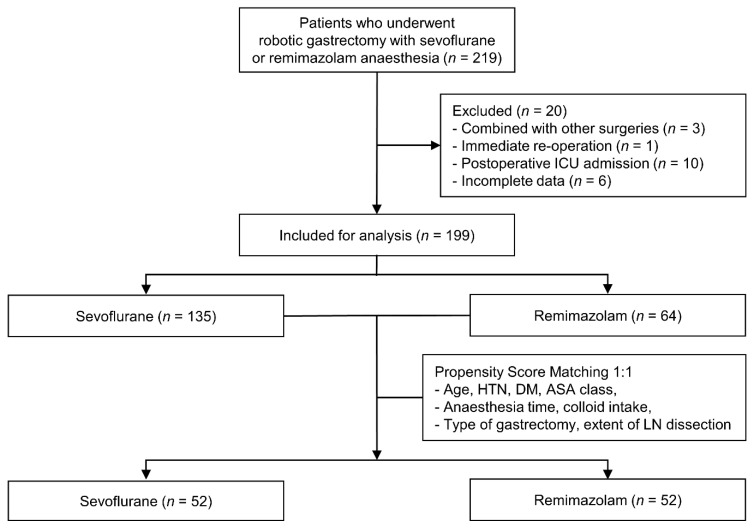
Flow diagram of the study. ICU, intensive care unit; HTN, hypertension; DM, diabetes mellitus; ASA, American Society of Anesthesiologists; LN, lymph node.

We assessed the electronic medical records of 219 consecutive patients who underwent robotic gastrectomy performed by four surgeons with sevoflurane or remimazolam anesthesia from January to November 2021. Of the 219 eligible patients, those who underwent robotic gastrectomy in combination with other procedures (*n* = 3) or immediate emergent reoperation (*n* = 1), those admitted to the intensive care unit (ICU) following surgery (*n* = 10), and those with incomplete data (*n* = 6) were excluded. The remaining 199 patients were then stratified into two groups according to the anesthetic agent used. Propensity score matching (PSM) was performed to match patients in the sevoflurane and remimazolam groups at a ratio of 1:1, using the following eight covariates: age, history of hypertension, history of diabetes mellitus (DM), American Society of Anesthesiologists (ASA) physical status, anesthesia time, colloid intake, type of gastrectomy, and extent of lymph node dissection (Figure 1).

### 2.2. Anesthetic Management

The type of anesthetic agent used was selected at the discretion of the attending anesthesiologist. In the sevoflurane group, anesthesia was induced via a bolus dose of propofol (1.0–2.0 mg·kg^−1^) and remifentanil infusion (0.05–0.1 ug·kg^−1^·min^−1^), followed by maintenance with sevoflurane (age-adjusted minimal end-tidal alveolar concentration: 0.8–1.0) combined with remifentanil infusion. In the remimazolam group, remimazolam (Byfavo injection Hana Pharm. Co., Ltd., Seoul, Korea) was administered via continuous infusion at rates of 6 mg·kg^−1^·h^−1^ and 1–2 mg·kg^−1^·h^−1^ for anesthesia induction and maintenance, respectively, and remifentanil was infused at doses similar to those described in a previous study [12].

In the operating room, the required devices for monitoring electrocardiographic parameters, pulse oximetry, and noninvasive blood pressure were applied, along with a SedLine^®^ electroencephalography sensor (Masimo Corp., Irvine, CA, USA) for monitoring the Patient State Index (PSI) and a peripheral nerve stimulator for monitoring neuromuscular blockade. An esophageal Doppler probe was inserted into the distal third of the esophagus, and it was connected to the esophageal Doppler monitor (CardioQ-ODM; Deltex Medical Ltd., Chichester, UK) to identify optimal blood flow from the descending aorta in order to measure cardiac output and cardiac index (CI). After 0.1 mg of glycopyrrolate was administered as premedication, anesthesia was induced and maintained as described above. After loss of consciousness was confirmed, 0.6–1.0 mg·kg^−1^ rocuronium was infused prior to endotracheal intubation to maintain neuromuscular relaxation during pneumoperitoneum, with a target train-of-four of 0–2 [13,14]. The attending anesthesiologist adjusted the doses of anesthetic agents to maintain PSI values of 40–60. In accordance with our institution’s protocol [15,16], the mean blood pressure (MBP) and heart rate (HR) were maintained within 20% of baseline values, and hypotension (20% reduction from baseline value or MBP < 60 mmHg) was managed using ephedrine (4–6 mg increments) or phenylephrine (20 μg increments or continuous infusion), as well as norepinephrine when necessary. Nicardipine was used to manage hypertension. At the end of the operation, neuromuscular blockade was reversed using sugammadex (1.0–2.0 mg·kg^−1^). During the emergence phase, sevoflurane vaporizers were turned off, and remimazolam infusion was discontinued with simultaneous administration of 0.2–0.3 mg of flumazenil (Flunil^®^ Bukwang Pharm Co., Ltd., Seoul, Korea). Additional doses of flumazenil were administered in 0.1 mg increments when the patient was not fully awake or unable to follow instructions, with the total dose not exceeding 0.5 mg. Spontaneous eye opening and the ability to follow instructions were confirmed before removal of the endotracheal tube. After the stability of vital signs and spontaneous respiration was confirmed, the patient was sent to the recovery room (RR).

### 2.3. Data Collection and Outcomes

Baseline characteristics including age, sex, body mass index (BMI), ASA physical status, and comorbidities were recorded for each patient. Surgical records were reviewed to obtain data regarding the type of gastrectomy, type of reconstruction, extent of lymph node dissection, and tumor–node–metastasis (TNM) stage. The following intraoperative variables were also recorded: operation time and anesthesia time, input and output information during the procedure (e.g., total fluid volume, colloid intake, blood loss, and urine output), anesthetic intake (e.g., total amount of remifentanil infused, total amount of remimazolam used, and the average concentration of sevoflurane during anesthesia), amount of sufentanil used for rescue analgesia, doses of flumazenil administered, and extubation time (time from anesthetic discontinuation to the time of extubation). Intraoperative hemodynamic variables such as MBP, HR, cardiac index (CI), and pulse-pressure variation (PPV) were assessed. Lastly, administered amounts, incidence, and use of ephedrine, phenylephrine, and norepinephrine during surgery were investigated. Postoperative recovery was evaluated on the basis of pain levels as assessed using a numeric rating scale, rescue analgesics administered, and the incidence of postoperative nausea and vomiting (PONV), postoperative shivering, delirium, re-sedation, and anxiety in the RR. We also analyzed Aldrete scores obtained 10 min after RR admission and before discharge from the RR, as well as the duration of postoperative hospitalization.

The primary outcome was the difference in use of intraoperative vasopressors between the groups. Secondary outcomes comprised group differences in the incidence of vasopressor use, intraoperative administered amounts of vasopressors, and intraoperative hemodynamic variables including MBP, HR, CI, and PPV.

### 2.4. Statistical Analysis

Continuous variables are presented as medians (first quartile, third quartile), while categorical variables are presented as numbers (percentage). The Mann–Whitney U-test, chi-square test, and Fisher’s exact test were used to compare data between the two groups. Logistic regression analysis was performed to evaluate the association between anesthesia type and outcomes. Variables with *p*-values < 0.05 in the univariable analysis were included in the multivariable model.

PSM was performed to reach a balanced distribution of patient characteristics between the two groups, and then to mimic a randomized patient composition. The propensity score was calculated using a logistic regression model including group as the dependent variable and the following potential confounding factors as independent variables: age, anesthesia time, hypertension, DM, ASA class, colloid intake, type of gastrectomy, and extent of lymph node dissection. PSM was performed using the nearest-neighbor method with a caliper of 0.05 and a 1:1 ratio of treated units to control units. The variables in the matched dataset were considered balanced between the groups when the standardized mean difference (SMD) was below 0.1. A conditional logistic regression model was employed to estimate odd ratios (ORs) for the remimazolam group for several outcomes in the matched dataset.

A linear mixed-model analysis (LMM) was conducted to assess the effects of group, time, and the group-by-time interaction for repeated-measures data, in which the compound symmetry covariance structure was imposed to address the within-subject effect. LMM analyses were adjusted for age, anesthesia time, hypertension, DM, ASA class, colloid intake, type of gastrectomy, and extent of lymph node dissection. The statistical analysis was conducted using SAS version 9.4 (SAS Institute, Cary, NC, USA), and *p*-values less than 0.05 were considered significant.

## 3. Results

### 3.1. Patient Characteristics

**Figure 2:**
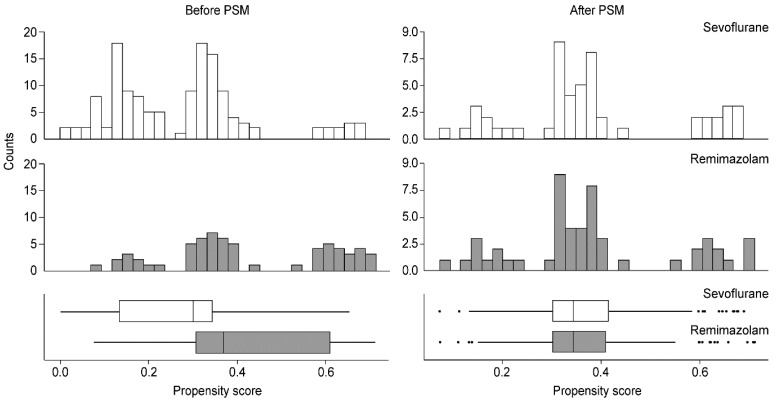
Distribution of propensity scores before and after matching. PSM, propensity score matching.

Prior to PSM, the sevoflurane and remimazolam groups included 135 and 64 patients, respectively. Older age, ASA class III, DM, and D2 lymph node dissection were more frequent in the sevoflurane group than in the remimazolam group. Other variables were comparable between the groups. After PSM, each group included 52 patients, and no significant differences in baseline characteristics were observed between the groups (Table 1). Figure 2 shows the distributions of propensity scores in both groups, which were similar after matching.

### 3.2. Perioperative Variables

Intraoperative and postoperative recovery variables are shown in Table 2. Total remifentanil administration and fluid intake were significantly greater, while time to extubation was significantly shorter in the remimazolam group than in the sevoflurane group. All patients in the remimazolam group received flumazenil for reversal at a median dose of 0.3 (0.3, 0.3) mg. The analysis of recovery variables demonstrated a longer RR stay, greater frequency of postoperative shivering and anxiety, and higher number of patients with Aldrete scores of 10 at 10 min after RR admission in the remimazolam group than in the sevoflurane group. However, Aldrete scores before discharge from the RR were lower in the remimazolam group than in the sevoflurane group. After PSM, there were no differences in the incidence of PONV, re-sedation, delirium, or anxiety between the groups; however, the incidence of postoperative shivering remained higher in the remimazolam group. The length of postoperative hospital stay was similar in the remimazolam and sevoflurane groups both before (5 [4, 5] vs. 5 [4, 5] days, *p* = 0.198) and after (5 [4, 5] vs. 5 [4, 5] days, *p* = 0.836) PSM.

### 3.3. Vasopressor Use

**Figure 3:**
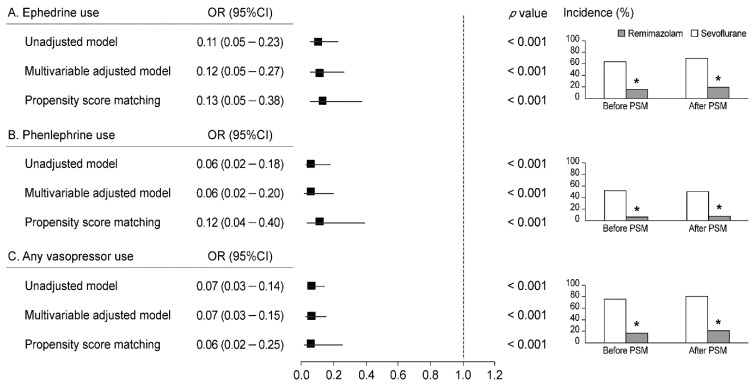
Forest plot and bar graph depicting the association of (**A**) ephedrine use, (**B**) phenylephrine use, and (**C**) any vasopressor use with remimazolam anesthesia vs. sevoflurane anesthesia. OR, odds ratio; 95% CI, 95% confidence interval; PSM, propensity score matching. * *p* < 0.05 vs. sevoflurane group.

Remimazolam anesthesia was associated with a significantly less frequent use of ephedrine (OR: 0.13, 95% CI: 0.05–0.38, *p* < 0.001), phenylephrine (OR: 0.12, 95% CI: 0.04–0.40, *p* < 0.001), and vasopressors in general (OR: 0.06, 95% CI: 0.02–0.25, *p* < 0.001) (Figure 3). In addition, the incidence of ephedrine, phenylephrine, and any vasopressor use was significantly lower in the remimazolam group than in the sevoflurane group both before and after PSM (all *p* < 0.001, Figure 3). The median doses of ephedrine and phenylephrine administered were significantly lower in the remimazolam group (0 [0, 0]) mg and 0 [0, 0] μg) than in the sevoflurane group (4 [0, 8] mg and 250 [0, 2100] μg) (all *p* < 0.001).

### 3.4. Intraoperative Hemodynamics

**Figure 4:**
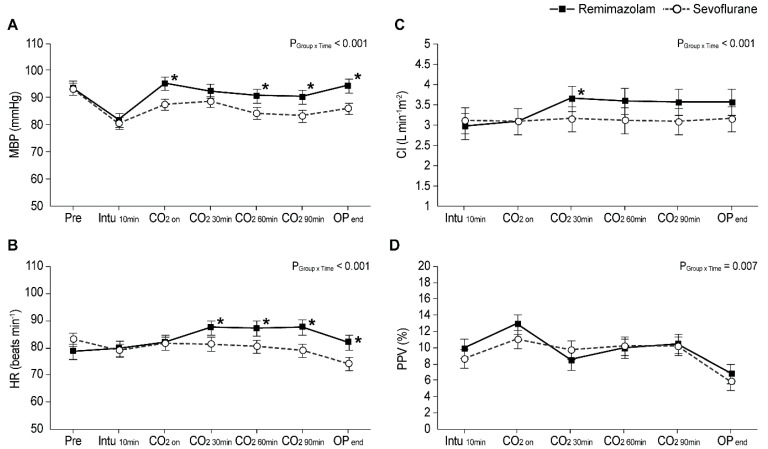
Intraoperative hemodynamic changes in the (**A**) mean blood pressure (MBP), (**B**) heart rate (HR), (**C**) cardiac index (CI), and (**D**) pulse-pressure variation (PPV). Values represent the estimated means from linear mixed models with standard error. PRE, pre-induction; Intu 10 min, 10 min after intubation; CO_2_ ON, initiation of CO_2_ pneumoperitoneum combined with head-up positioning; CO_2 30min_, 30 min after initiation of CO_2_ pneumoperitoneum; CO_2 60min_, 60 min after initiation of CO_2_ pneumoperitoneum; CO_2 90min_, 90 min after initiation of CO_2_ pneumoperitoneum; OP end, end of operation. * Bonferroni-corrected *p* < 0.05 vs. sevoflurane group.

Figure 4 shows the intraoperative changes in MBP, HR, CI, and PPV in both groups. Intraoperative MBP was significantly higher in the remimazolam group than in the sevoflurane group immediately after CO_2_ insufflation, 60 and 90 min after CO_2_ insufflation, and at the end of the operation (Bonferroni-corrected *p*: <0.001, 0.008, 0.003, and <0.001, respectively). From 30 min after CO_2_ insufflation until the end of the operation, HR was significantly higher in the remimazolam group than in the sevoflurane group (Bonferroni-corrected *p*: 0.015, 0.006, <0.001, and <0.001, respectively). Before adjustment, the CI was significantly higher in the remimazolam group than in the sevoflurane group at 30 min, 60 min, and 90 min after CO_2_ insufflation and at the end of the operation (Bonferroni-corrected *p*: 0.002, 0.004, 0.003, and 0.014, respectively). However, in the post-adjustment LMM analysis, the CI remained significantly higher in the remimazolam group only at 30 min after insufflation (3.6 vs. 3.2 L·min^−1^·m^−2^, Bonferroni-corrected *p* = 0.048) and was comparable between the groups at all other timepoints. Although the LMM analysis revealed a pattern indicating a significant difference in PPV between the groups (*p* group × time = 0.007), a post hoc analysis with Bonferroni correction revealed no significant differences in PPV between the groups at any timepoint.

## 4. Discussion

The present study represents the first case-matched retrospective comparison of intraoperative hemodynamics between patients receiving remimazolam and sevoflurane anesthesia during robotic gastrectomy for gastric cancer. Remimazolam anesthesia was associated with a significantly less frequent use of vasopressors than sevoflurane anesthesia. In addition, a significantly lower incidence of vasopressor use, as well as significantly lower administered doses of vasopressors, was observed with remimazolam anesthesia compared to sevoflurane anesthesia. Furthermore, MBP was maintained at significantly higher levels during CO_2_ pneumoperitoneum in the remimazolam group than in the sevoflurane group despite relatively lower doses of vasopressor administration in the former.

According to our results, the anesthetic agent used for robotic gastrectomy exerts a profound effect on hemodynamic stability and the requirement for vasopressors. Previous studies have suggested that CO_2_ pneumoperitoneum and the head-up reverse Trendelenburg position work in tandem to lower the cardiac output [4]. In this context, general anesthesia with remimazolam may attenuate the effects of pneumoperitoneum, hypercarbia, and the reverse Trendelenburg position when compared with sevoflurane anesthesia, allowing for better maintenance of hemodynamic stability. Alternatively, sevoflurane may accentuate the hemodynamic effects of CO_2_ insufflation and positioning, thereby lowering MBP during pneumoperitoneum when compared with remimazolam. However, it is unlikely that remimazolam would lessen the actual mechanical compression due to CO_2_ insufflation or attenuate the hypercarbia and decrease in preload due to head-up positioning. Another, more likely hypothesis is that remimazolam enables better maintenance of hemodynamic stability without exerting any direct influence on the intraabdominal pressure or the effects of head-up positioning. To reiterate, a hypothesis could be made that, aside from surgical components such as positioning and pneumoperitonum, the choice of anesthetics could have a significant effect on hemodynamic stability during robotic gastrectomy, perhaps more so than previously thought. This hypothesis can be further supported by the fact that the study comparing remimazolam and propofol, which included various surgical procedures without pneumoperitoneum or reverse Trendelenburg positioning, was also able to demonstrate that vasopressor requirements were lower in patients receiving remimazolam [12]. Ultimately, further prospective studies will be needed to examine the mechanism via which remimazolam better maintains hemodynamic stability.

The current results support our hypothesis that MBP is better maintained with remimazolam anesthesia than with sevoflurane anesthesia. This significant difference in MBP between groups was maintained throughout most of the surgery except at 30 min into pneumoperitoneum, and this difference in MBP between groups occurred despite more frequent use and higher doses of vasopressors administered in the sevoflurane group. In a study examining the hemodynamics during laparascopic cholecystectomy, the authors reported a partial return of hemodynamics at 30 min into pneumoperitoneum, in which they concluded was due to the vasodilating properties of the anesthetics or the natural history of the hemodynamic changes [17]. This could explain why, in our results, the difference in MBP was not significant at the 30 min timepoint, although between-group differences in MBP persisted throughout most of the procedure. Examining the hemodynamic mechanism is beyond the scope of this study, and a well-designed, prospective study in the future might provide better insights. After PSM, the CI was significantly higher in the remimazolam group than in the sevoflurane group at 30 min into pneumoperitoneum, although the difference was not significant thereafter. Notably, significantly more patients in the sevoflurane group received vasopressors, which may have led to an increase in the CI. Although CI values at 60 min into pneumoperitoneum and beyond were comparable between the groups, one can argue that remimazolam anesthesia better maintains both MBP and CI, considering the lower doses of vasopressors administered in the remimazolam group. Moreover, the differences in MBP and HR persisted after desufflation, following which the return of hemodynamic stability is thought to be immediate in healthy individuals. Further prospective studies are required to determine how long these differences persist and whether they are associated with any clinical benefits.

The amount of remifentanil administered was significantly higher in the remimazolam group than in the sevoflurane group, which is consistent with previous results [12,18]. We do not believe that this difference in remifentanil dose confounded our results. Firstly, as an opioid, remifentanil is more likely to decrease HR than sevoflurane, and the HR observed in the remimazolam group was significantly higher than that observed in the sevoflurane group, suggesting that opioid/analgesic effects did not substantially influence hemodynamic results in our study. This increase in HR is consistent with that observed in a previous study in which remimazolam alone was administered to healthy volunteers [10]. Secondly, if equal amounts of remifentanil were to be administered in the sevoflurane group, it is reasonable to assume that vasopressor requirements during sevoflurane anesthesia would have been considerably higher than those observed in our study. Additionally, the use of remimazolam as the hypnotic component is what allows for higher doses of remifentanil administration, and it would be difficult and impractical to separate the effects of remimazolam and remifentanil or to assume that one component per se is responsible for the difference in hemodynamics.

Fluid intake was also significantly greater in the remimazolam group than in the sevoflurane group. It is important to note that remimazolam preparations for infusion were at a concentration of 1 or 2 mg·mL^−1^. Previous reports have indicated that precipitation can occur at lower infusion rates [19], and it is most likely that a minimum infusion rate was maintained to prevent this scenario. However, the difference in fluid intake is unlikely to explain our results, as there was no significant difference in the PPV between the groups at any point during anesthesia, suggesting that volumes were equal and adequate in the remimazolam and sevoflurane groups.

Regarding postoperative delirium, there were no documented case of delirium in any of the patients included in this study. Further prospective studies, designed to actively screen for delirium and more accurately assess incidence in the postoperative period would be needed to make any definite conclusions. While none of our patients experienced seizures or extreme anxiety, a significant number of patients in the remimazolam group experienced postoperative shivering compared to that in the sevoflurane group. Results of a systematic meta-analyses revealed that flumazenil use was associated with increased shivering and chills [20]; therefore, flumazenil could be the most likely explanation for the increased shivering seen in our results. All patients who experienced shivering were treated with an intravenous dose of pethidine, and all patients experienced resolution of symptoms before discharge from the RR. We believe that further prospective studies are required to investigate the reason for this increase in postoperative shivering. Remimazolam is also the only hypnotic used for general anesthesia that has an antagonizing agent [6]. Of note, all patients in the remimazolam group received flumazenil for reversal during emergence. Although this was not the primary outcome of our study and further studies will be needed, these results could support the argument for the use of flumazenil for reversal in patients receiving remimazolam for general anesthesia [20].

This study had some limitations. First of all, this study was a retrospective study. Exploration of the mechanisms in which remimazolam maintains better hemodynamic stability and examination of the association between remimazolam and postoperative shivering or anxiety were beyond the scope of this study; hence, further prospective studies will be needed. However, our study groups were well matched on the basis of propensity scores, and we believe that our results regarding the hemodynamic benefits of remimazolam as the main hypnotic during general anesthesia are compelling. Secondly, this study was conducted at a single institution, and differences in case volume and surgical experience were likely to influence factors such as the duration of surgery, which may limit the generalizability of our results. Thirdly, because it would have required for a prospective design with a standardized protocol, we could not control for the degree of deep neuromuscular blockade, which is known to affect the quality and ease of laparascopic surgery [21]. Lastly, most patients included in our study had an ASA physical status of II or III, and the results may be different for different populations. For example, no patients in our study had significant liver disease or end-stage renal failure, both of which are known to affect the pharmacokinetic profile of remimazolam [22].

## 5. Conclusions

In conclusion, our findings suggest that remimazolam enables better maintenance of hemodynamic stability than sevoflurane when used for general anesthesia. Thus, remimazolam anesthesia may be beneficial for patients who are expected to experience hypotension due to the combined effects of CO_2_ pneumoperitoneum and the head-up position utilized during robotic gastrectomy.

## Figures and Tables

**Table 1:** Patient characteristics before and after propensity score matching.

	Before Propensity Score Matching				After Propensity Score Matching			
	Group S (*n* = 135)	Group R (*n* = 64)	*p*-Value	SMD	Group S (*n* = 52)	Group R (*n* = 52)	*p*-Value	SMD
Age (years)	60 (52, 66)	55 (46, 62.5)	0.035	0.298	57.5 (47, 66)	57 (46, 64)	0.643	0.046
Male sex	76 (56%)	38 (59%)	0.681	0.062	47 (90%)	48 (92%)	0.201	0.234
BMI (kg·m^−2^)	22 (20, 25)	23 (21, 24)	0.386	0.035	22 (20, 24)	23 (22, 24)	0.132	0.197
ASA physical status			0.006	0.437			0.739	0.050
II	89 (66%)	54 (84%)			43 (83%)	42 (81%)		
III	46 (34%)	10 (16%)			9 (17%)	10 (19%)		
Comorbidities								
Hypertension	46 (34%)	17 (27%)	0.287	0.164	37 (71%)	36 (69%)	0.818	0.042
Diabetes mellitus	24 (18%)	4 (6%)	0.029	0.360	5 (10%)	4 (8%)	0.317	
Carotid artery stenosis	7 (5%)	3 (5%)	>0.999	0.023	3 (6%)	3 (6%)	>0.999	<0.001
Old CVA	3 (2%)	0 (0%)	0.552	0.213	0 (0%)	0 (0%)	-	<0.001
COPD	6 (4%)	4 (6%)	0.729	0.080	2 (4%)	3 (6%)	0.654	0.090
Angina	3 (2%)	0 (0%)	0.552	0.213	0 (0%)	0 (0%)	-	<0.001
Atrial fibrillation	6 (4%)	1 (2%)	0.432	0.169	1 (2%)	1 (2%)	>0.999	<0.001
CAD	4 (3%)	3 (5%)	0.683	0.090	0 (0%)	3 (6%)	0.083	0.350
Type of gastrectomy			0.618	0.147			0.921	0.139
Subtotal	106 (79%)	50 (78%)			42 (81%)	41 (79%)		
Proximal subtotal	10 (7%)	7 (11%)			4 (8%)	6 (12%)		
Total	19 (14%)	7 (11%)			6 (11%)	5 (9%)		
Type of reconstruction			0.302	0.296			0.731	0.161
Billroth I	60 (44%)	35 (55%)			25 (48%)	28 (53%)		
Billroth II	39 (29%)	12 (19%)			15 (29%)	12 (23%)		
Double-tract	10 (7%)	7 (11%)			5 (10%)	6 (12%)		
Roux-en-Y	26 (19%)	10 (15%)			7 (13%)	6 (12%)		
Extent of LN dissection			0.004	0.464			>0.999	<0.001
D1	82 (61%)	52 (81%)			40 (77%)	40 (77%)		
D2	28 (21%)	12 (19%)			12 (23%)	12 (23%)		
TNM stage			0.105	0.406			0.543	0.189
I	82 (61%)	49 (77%)			39 (75%)	39 (75%)		
II	28 (21%)	11 (17%)			7 (13%)	9 (17%)		
III	9 (7%)	2 (3%)			2 (%)	2 (4%)		
IV	15 (11%)	2 (3%)			4 (8%)	2 (4%)		

Values are presented as the median (first quartile, third quartile) or number of patients (%). SMD: standardized mean difference, BMI: body mass index, ASA: American Society of Anesthesiologists, CVA: cerebrovascular accident, COPD: chronic obstructive pulmonary disease, CAD: coronary artery disease, LN: lymph node, TNM: tumor–node–metastasis.

**Table 2:** Intraoperative and recovery room variables before and after propensity score matching.

	Before Propensity Score Matching				After Propensity Score Matching			
	Group S (*n* = 135)	Group R (*n* = 64)	*p*-Value	SMD	Group S (*n* = 52)	Group R (*n* = 52)	*p*-Value	SMD
Intraoperative variables								
Operation time (min)	187 (150, 238)	171 (140, 217)	0.078	0.273	173 (144, 208)	173 (136, 218)	0.913	0.030
Anesthesia time (min)	215 (180, 270)	204 (175, 247)	0.122	0.270	202 (170, 232)	212 (172, 250)	0.757	0.063
Fluid intake (mL)	1500 (1200, 1900)	1850 (1550, 2300)	<0.001	0.534	1425 (1150, 1650)	1900 (1575, 2400)	<0.001	0.883
Colloid intake (mL)	0 (0, 500)	500 (0, 500)	0.060	0.265	500 (0, 500)	500 (0, 500)	>0.999	<0.001
Transfusion	0 (0%)	0 (0%)	-	<0.001	0 (0%)	0 (0%)	-	<0.001
Blood loss (mL)	35 (20, 90)	40 (12, 90)	0.656	0.080	29 (20, 50)	45 (16, 90)	0.176	0.260
Urine output (mL)	180 (120, 290)	180 (135, 312)	0.711	0.070	187 (125, 285)	180 (132, 307)	0.736	0.090
Sevoflurane (%)	1.7 (1.6, 1.8)	-	-	-	1.7 (1.6, 1.8)	-	-	-
Remimazolam (mg)	-	316 (244, 368)	-	-	-	322 (244, 391)	-	-
Remifentanil (µg)	800 (600, 1000)	1445 (987, 2030)	<0.001	1.260	700 (600, 900)	1405 (1023, 1990)	<0.001	1.280
Sufentanil rescue (µg)	6 (6, 7)	6 (6, 7)	0.413	0.142	6 (5, 7)	6 (6, 7)	0.767	0.040
Fumazenil (mg)		0.3 (0.3, 0.3)				0.3 (0.3, 0.3)		
Extubation time (sec)	420 (300, 600)	115 (69, 172)	<0.001	2.055	360 (300, 540)	115 (75, 180)	<0.001	1.883
Recovery room variables								
Time in RR (min)	37 (30, 45)	40 (35, 50)	<0.001	0.480	35 (30, 40)	40 (35, 50)	0.005	0.689
Numeric rating scale	3 (3, 3)	3 (2, 5)	0.338	0.178	3 (3, 3)	3 (2, 5)	0.259	0.218
Rescue analgesics (µg)	25 (0, 50)	45 (0, 50)	0.118	0.244	35 (0, 50)	50 (0, 50)	0.176	0.307
PONV	6 (4%)	1 (2%)	0.433	0.169	5 (10%)	1 (2%)	0.103	0.334
Shivering	4 (3%)	12 (19%)	<0.001	0.525	1 (2%)	10 (19%)	0.007	0.586
Delirium	0 (0%)	0 (0%)	-	<0.001	0 (0%)	0 (0%)		<0.001
Re-sedation	0 (0%)	2 (3%)	0.102	0.254	0 (0%)	1 (2%)	0.317	0.198
Anxiety	0 (0%)	3 (5%)	0.032	0.314	0 (0%)	3 (6%)	0.083	0.350
Aldrete score 10 min	9 (9, 9)	9 (9, 10)	0.022	0.313	9 (9, 9)	9 (9, 10)	0.002	0.710
Before discharge	10 (10, 10)	10 (9, 10)	<0.001	<0.001	10 (10, 10)	10 (9, 10)	<0.001	0.892

Values are presented as the median (first quartile, third quartile) or number of patients (%). SMD: standardized mean difference, RR: recovery room, PONV: postoperative nausea and vomiting.

## Data Availability

The datasets used and/or analyzed during the current study are available from the corresponding author on reasonable request.
